# Ligand dimensions are important in controlling NK-cell responses

**DOI:** 10.1002/eji.201040335

**Published:** 2010-04-28

**Authors:** Joanna Brzostek, Jian-Guo Chai, Friedemann Gebhardt, Dirk H Busch, Rui Zhao, P Anton van der Merwe, Keith G Gould

**Affiliations:** 1 Department of Immunology, Wright-Fleming Institute, Imperial College LondonLondon, UK; 2 Department of Immunology, Division of Medicine, Imperial College LondonLondon, UK; 3 Institute for Microbiology, Immunology and Hygiene, Technical University MunichMunich, Germany; 4 Laboratory of Lymphocyte Development and Signalling, The Babraham InstituteCambridge, UK; 5 Sir William Dunn School of Pathology, University of OxfordOxford, UK

**Keywords:** Cell surface molecules NK cells, Cellular activation, NK-cell ligands

## Abstract

Size-dependent protein segregation at the cell–cell contact interface has been suggested to be critical for regulation of lymphocyte function. We investigated the role of ligand dimensions in regulation of mouse NK-cell activation and inhibition. Elongated forms of H60a, a mouse NKG2D ligand, were generated and expressed stably in the RMA cell line. RMA cells expressing the normal size H60a were lysed efficiently by both freshly isolated and IL-2 stimulated C57BL/6 mouse-derived NK cells; however the level of lysis decreased as the H60a ligand size increased. Importantly, H60a elongation did not affect NKG2D binding, as determined by soluble NKG2D tetramer staining, and by examining NK-cell target cell conjugate formation. CHO cells are efficient at activating NK cells from C57BL/6 mice, and expression of a single chain form of H-2K^b^, a ligand for the mouse inhibitory receptor Ly49C, strongly inhibited such activation of Ly49C/I positive NK cells. Elongation of H-2K^b^ resulted in decreased inhibition of both lysis and IFN-γ production by NK cells. These results establish that small ligand dimensions are important for both NK-cell activation and inhibition, and suggest that there are shared features between the mechanisms of receptor triggering on different types of lymphocytes.

## Introduction

Receptor–ligand interactions are of fundamental importance in the regulation of lymphocyte function. Extensive information is now available on structural features of lymphocyte receptors, and the signalling pathways they initiate. However, the mechanisms of lymphocyte receptor triggering, defined as the sequence of molecular events that link receptor ligation to initiation of the signalling pathways, remain much less well characterized. It has been proposed that the relatively small dimensions of receptor-ligand pairs are critical for lymphocyte receptor triggering, as they allow size-dependent protein segregation at the contact interface between cells 1.

The importance of ligand dimensions for CD8^+^ T-cell activation has been demonstrated by experimental elongation of MHC class I molecules, which resulted in a marked reduction of TCR-dependent signalling and T-cell effector function 2,3. A similar dependence on ligand size has been demonstrated for T cells expressing a chimeric TCR 4. The significance of size-dependent segregation of proteins during lymphocyte interactions has also been shown by elongation of CD48, a ligand for the T-cell co-receptor CD2 5,6, and by experimental reduction in size of the large ectodomain of CD45 7, both of which led to a reduction in T-cell activation. A requirement for optimal ligand dimensions in T-cell activation is therefore of importance when manipulating TCR and their ligands for possible therapeutic purposes. For example, T cells expressing a chimeric TCR that recognises a membrane-distal epitope on CD22 have reduced effector functions on CD22 positive target cells, as compared with T cells expressing a TCR recognising a membrane-proximal epitope 8. Because CD22 is upregulated on B-cell lymphomas, this higher level of expression allows the T cells with the TCR recognising the membrane-distal epitope on CD22 to lyse lymphoma cells, while sparing normal B cells 8.

We wanted to investigate whether ligand dimensions are also important in NK-cell activating and inhibitory receptor triggering. NK cells are the third largest lymphocyte population and play a key role in the recognition and elimination of viruses and tumours. Identification of abnormal cells, using sets of activating and inhibitory receptors, initiates NK-cell-mediated killing and cytokine production. NK-cell activating receptors, such as NKG2D and Ly49H, recognise stress-induced ligands or virus-derived molecules, respectively. Inhibition of NK-cell function is mediated by inhibitory receptors such as Ly49C and CD94/NKG2A that interact with MHC class I molecules. Ligation of NK-cell receptors leads to tyrosine phosphorylation of receptor-associated cytoplasmic activating (ITAM) or inhibitory (ITIM) motifs, initiating the relevant downstream signalling pathways. A single NK cell expresses several different activating and inhibitory receptors, and the outcome of a NK cell–target cell interaction depends on the balance between activating and inhibitory signalling.

In order to study the role of ligand dimensions in NK-cell activation, we have generated elongated forms of the MHC class I homologue H60a, a mouse NKG2D ligand 9–11 and expressed them stably in the RMA cell line. As inhibitory ligands for mouse NK cells we have used the MHC class I H-2K^b^ molecule, a ligand for the Ly49C receptor, and Qa1^b^, ligand for CD94/NKG2A, together with elongated versions of these ligands. For ligand elongation we inserted two, three or four Ig domains from human CD2, mouse CD22 containing a mutation that makes it non-functional and human CD4, respectively. These were the same inserts used in our previous work on the role of ligand dimensions in T-cell activation 2,3, and were selected because they were likely to be inert for mouse NK cells. Our results show that small ligand dimensions are important for both NK-cell activation and inhibition. This finding extends the significance of ligand-receptor dimensions to an additional type of lymphocyte, emphasising that similar principles may be important in controlling receptor-mediated responses during cell–cell interactions for all lymphocytes.

## Results

### Engineering elongated H60a molecules

**Figure 1:**
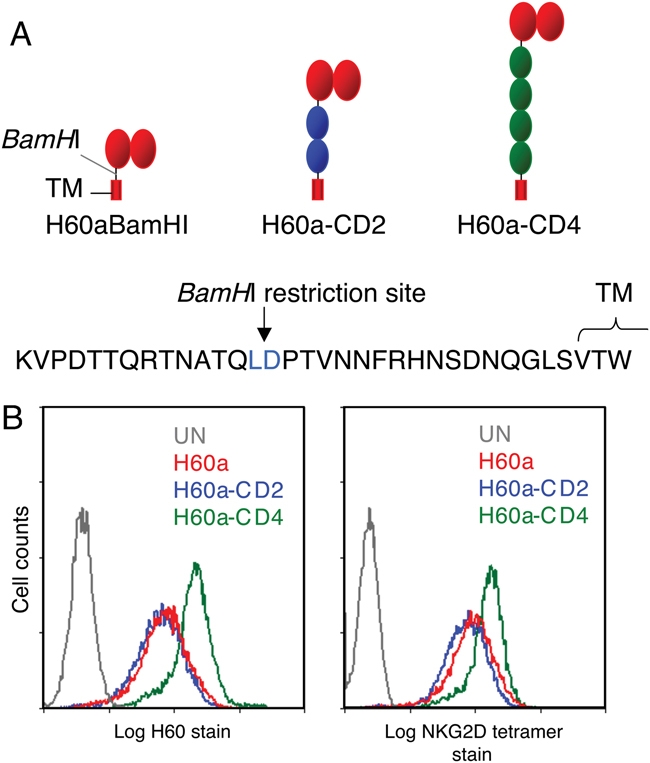
Elongated forms of H60a are expressed at the cell surface, and bind NKG2D. (A) Schematic representation of H60a and its elongated forms, created by insertion of additional Ig-like domains from human CD2 (H60a-CD2) or human CD4 (H60a-CD4) into the membrane-proximal region of H60a. The amino acid sequence of the relevant portion of H60a is shown below. The sequence in blue indicates the amino acids changed by addition of the *BamH*I restriction site, and the position of the transmembrane (TM) region is indicated. (B) Representative cell surface expression of the H60a constructs in FACS-sorted RMA cells, stained with either anti-H60 antibody or NKG2D tetramer. Untransfected RMA cells stained with the relevant reagent were used as a negative control.

In order to investigate the importance of ligand dimensions in NK-cell activation, we generated elongated forms of H60a, a NKG2D ligand that is a structural homologue of the α1α2 platform of MHC class I. Use of a NKG2D ligand offers distinct advantages, as all mouse NK cells express NKG2D 12, and the NKG2D signalling pathways are relatively well understood 13. Elongated forms of MHC class I 2,3 and CD48 5 were generated previously by insertion of independently-folding Ig domains into extracellular, membrane-proximal regions of these molecules. We used a similar approach to create elongated forms of H60a (Fig. 1A). A unique *BamH*I restriction site was introduced in the membrane-proximal region of the molecule (Fig. 1A), and inserts encoding two or four Ig domains derived from CD2 or CD4 were ligated in, to create constructs encoding H60a-CD2 and H60a-CD4 molecules, respectively. The constructs were stably expressed in the RMA cell line, with good cell surface expression as determined using anti-H60 antibody staining (Fig. 1B). The correct size of these engineered molecules was confirmed by western blotting (data not shown).

### Elongation of H60a does not alter NKG2D binding

**Figure 2:**
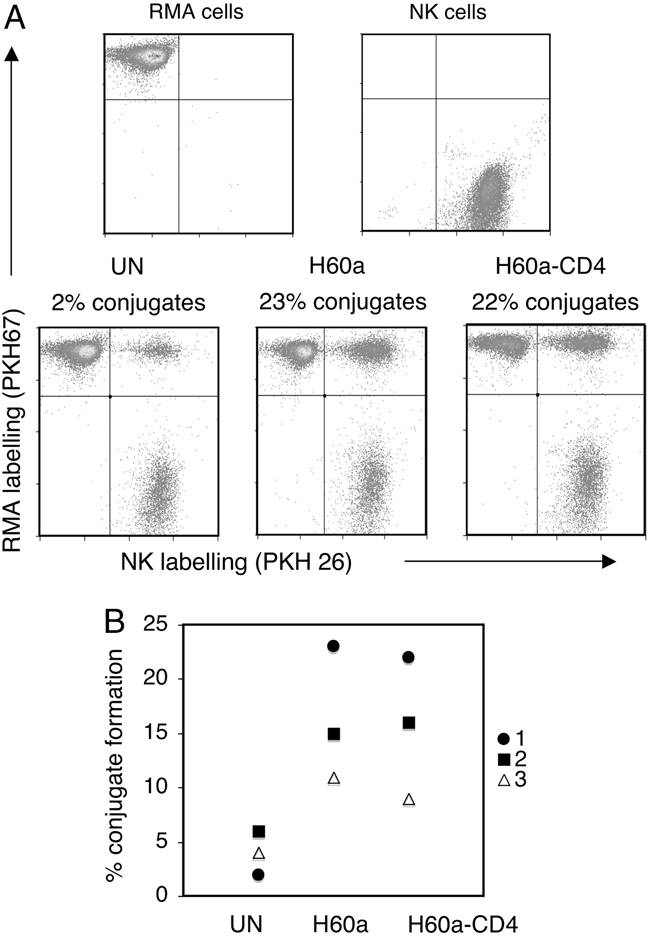
Elongated H60a supports efficient cell conjugate formation with NK cells. (A) Untransfected RMA cells (UN), or RMA cells expressing H60a or H60a-CD4, were labelled with PKH67 membrane dye, mixed with IL-2-expanded NK cells that had been labelled with PKH26 membrane dye and conjugate formation was then investigated using flow cytometry, as described in the *Materials and methods*. NK cells were pooled from groups of five mice. (B) Graph showing data from three independent experiments (1, 2 and 3), using different batches of NK cells. Experiment 1 is shown in (A).

Elongation of H60a by insertion of additional Ig domains may potentially interfere with the correct folding of the α1α2 platform of H60a, impairing NKG2D binding. To test this possibility, we investigated NKG2D binding to the standard and elongated H60a molecules using soluble NKG2D tetramer binding and a cellular conjugation assay. NKG2D tetramer stained the RMA cells expressing H60a, H60a-CD2 or H60a-CD4 strongly (Fig. 1B), reflecting exactly the pattern of antibody staining, strongly suggesting that elongation of H60a did not affect NKG2D binding. Moreover, titration of the NKG2D tetramer showed that the different H60a molecules had very similar affinities for soluble NKG2D (data not shown). The transfected cells shown in Fig. 1 were used to establish NKG2D binding to the different H60a ligands, but for subsequent use in functional experiments as targets, sorted cells with closely matched H60a ligand levels were used. We then probed the interaction between H60a and NKG2D in the context of cellular interactions using a conjugation assay. RMA cells expressing H60a, H60a-CD4 or untransfected RMA cells were labelled with PKH67 membrane dye and co-incubated for 10 min with IL-2 expanded B6 NK cells, labelled with PKH26 membrane dye. NK cells formed few conjugates with the untransfected RMA cells, and the conjugation increased when RMA cells expressed the standard H60a (Fig. 2). NK cells formed conjugates with RMA cells expressing H60a or the elongated H60a-CD4 equally well (Fig. 2), confirming that elongation of H60a does not alter NKG2D binding.

### Elongation of H60a reduces NK-cell lysis *in vitro*

**Figure 3:**
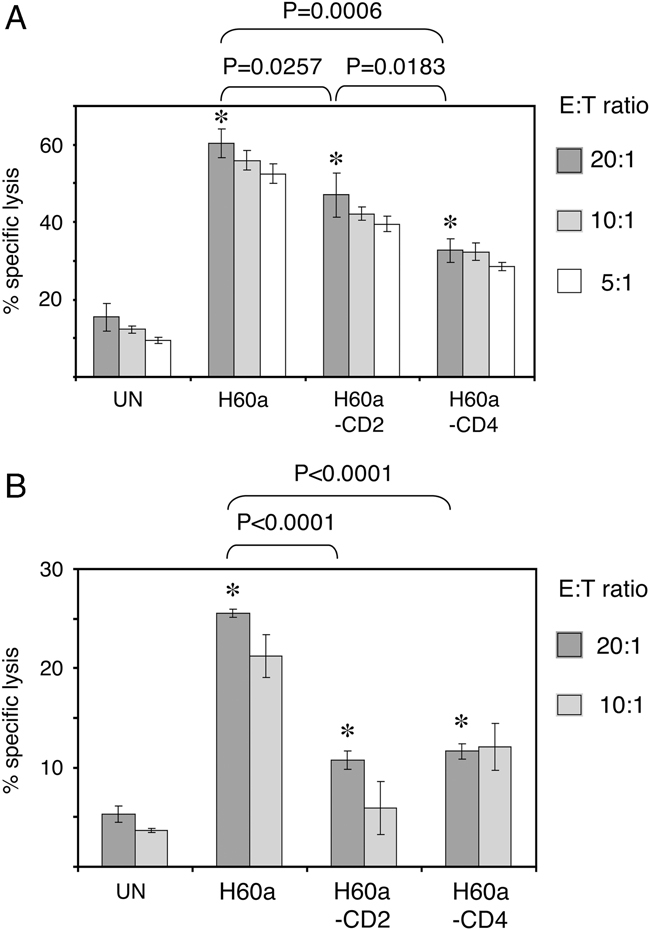
Elongation of H60a reduces NK cell lysis *in vitro*. Untransfected RMA cells (UN), RMA+H60a, RMA+H60a-CD2 and RMA+H60a-CD4 cells expressing similar levels of ligands were used as targets for (A) IL-2-expanded NK cells or (B) *ex vivo* B6 NK cells in ^51^Cr release assays at the indicated effector to target (E:T) ratios. Data show mean+SD (*n*=4). ^*^Two-tailed *p* values from unpaired *t* tests of significant difference are indicated for the data columns (20:1 E:T ratio). Data are representative of five (A) and two (B) independent experiments.

In order to investigate the functional consequences of ligand elongation on activation of NK cells, we used RMA cells expressing the unaltered or elongated forms of H60a as target cells in NK-cell chromium release cytotoxicity assays. Using IL-2 expanded NK cells as the effectors, we observed high levels of lysis of RMA cells expressing the WT H60a molecule (Fig. 3A). RMA cells expressing the elongated H60a molecules were lysed significantly less efficiently (Fig. 3A), with lysis levels of targets expressing H60a-CD4 reduced to approximately half those of targets expressing the standard H60a. In order to verify this finding, we repeated the killing assay using *ex vivo* NK cells as effectors. We observed lower levels of lysis overall, consistent with the unstimulated phenotype of freshly isolated NK cells (Fig. 3B). As observed with the IL-2 expanded NK cells, *ex vivo* NK cells lysed RMA+H60a cells significantly more efficiently than RMA cells expressing the elongated H60a molecules (Fig. 3B). These results demonstrated that H60a elongation reduces NKG2D-dependent activation of NK cells. We could not determine the effect of H60a elongation on the NK-cell cytokine response, because there was no IFN-γ secretion in response to the RMA cells expressing the standard form of H60a (data not shown), possibly reflecting relatively low levels of H60a cell surface expression.

### Elongation of H60a reduces NK-cell lysis *in vivo*

**Figure 4:**
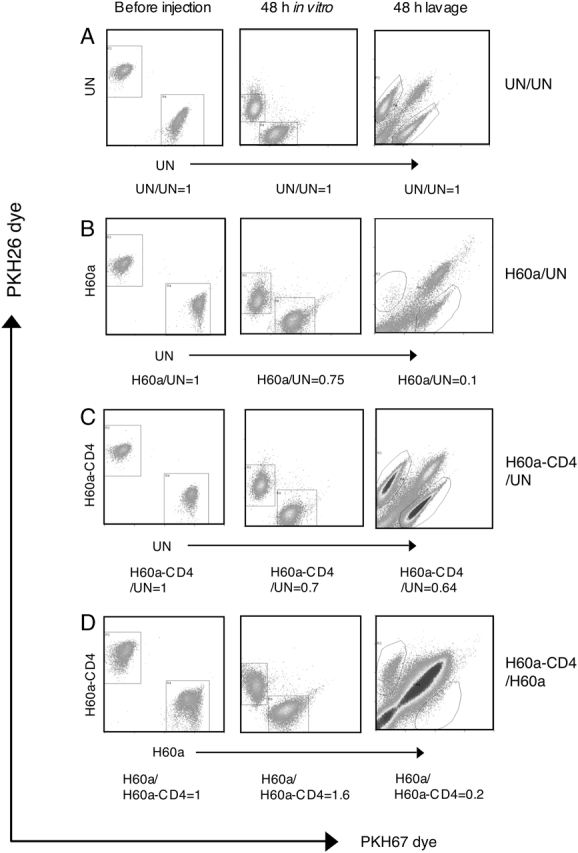
Elongation of H60a reduces lysis *in vivo*. The two indicated RMA cell populations were labelled separately with different membrane dyes, and injected at a 1:1 ratio i.p. into B6 mice. After 48 h, the peritoneal lavage was analysed by flow cytometry. Examples of the labelled cell populations analysed immediately before injection, after 48 h culture in vitro and in the peritoneal lavage are shown. (A) Untransfected RMA (UN) and untransfected RMA (UN). (B) RMA+H60a and untransfected RMA. (C) RMA+H60a-CD4 and untransfected RMA. (D) RMA+H60a-CD4 and RMA+H60a. Data are representative of four (A–C) and two (D) independent experiments, using two to four mice *per* group for each experiment.

Following the observed reduction of *in vitro* NK cells lysis of RMA cells expressing similar levels of the elongated forms of H60a molecule (Fig. 3), the physiological relevance of these findings was tested using an *in vivo* killing assay. Two cell populations, labelled separately with the membrane dyes PKH26 and PKH67, was injected i.p. into B6 mice at 1:1 ratio (see *Materials and methods*). After 48 h, the intraperitoneal lavage was analysed by flow cytometry. The input cell mixtures were analysed before injection, and an aliquot was also cultured for 48 h *in vitro*, to allow detection of any differences in dye loss between the two cell populations. When untransfected RMA cells were labelled separately with PKH26 and PKH67 and injected at 1:1 ratio, the original 1:1 ratio was observed 48 h later in the peritoneal lavage and after culture *in vitro* (Fig. 4A). This demonstrated that the labelling with the membrane dyes had no effect on RMA cell viability both *in vivo* and *in vitro*. When untransfected RMA cells were injected together with RMA cells expressing H60a, there was loss of RMA+H60a cells, relative to the untransfected RMA cells (Fig. 4B). Although there was some apparent loss of H60a cells relative to untransfected RMA cells in the *in vitro* culture, presumably due to a reduced rate of proliferation of the transfected cells, this was much less than the loss of H60a cells *in vivo*. Therefore, H60a expression on RMA cells stimulated lysis *in vivo*. When untransfected RMA cells were injected with RMA cells expressing the elongated H60a-CD4 molecule, some reduction of the H60a-CD4/RMA ratio was observed for both the peritoneal lavage and *in vitro* culture (Fig. 4C). However, the loss of RMA cells expressing the elongated H60a-CD4 molecules after the i.p. injection was much less than that of RMA cells expressing the unaltered H60a molecule, strongly suggesting that elongation of H60a reduces *in vivo* lysis. Differentially labelled H60a and H60a-CD4 cells were co-injected (Fig. 4D) in order to compare directly *in vivo* lysis of these two cell lines. In the peritoneal lavage, we observed loss of RMA cells expressing the unaltered H60a molecule relative to RMA cells expressing the H60a-CD4 molecule, even though RMA+H60a cells apparently proliferated more rapidly than RMA+H60a-CD4 cells *in vitro* (Fig. 4D), demonstrating directly that elongation of H60a reduces *in vivo* lysis. The 48 h lavage samples had a variable number of auto-fluorescent cells, which showed up on the diagonal of the flow cytometry plots (Fig. 4), which were probably macrophages. However, these cells did not interfere with the *in vivo* killing assay.

### Elongation of H60a reduces lysis by CD45^−/−^ NK cells

**Figure 5:**
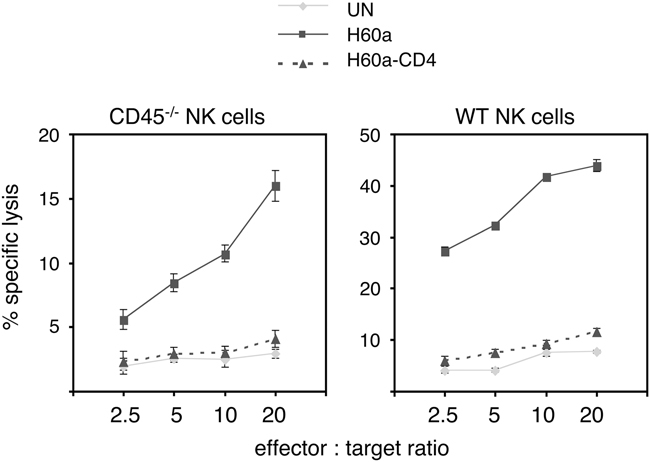
Elongation of H60a reduces lysis by both CD45^−/−^ and wild-type NK cells. Untransfected RMA cells (UN), RMA+H60a and RMA+H60a-CD4 cells were used as targets for IL-2-expanded CD45^−/−^ or WT NK cells in chromium release cytotoxicity assays. Data show mean+SD (*n*=4) and are representative of two independent experiments.

Elongation of H60a reduced NK-cell lysis *in vitro* and *in vivo* (Figs. 3 and 4); however the molecular mechanism of this effect is not known. It has been proposed that the relatively small dimensions of lymphocyte receptors and their ligands are critical for receptor triggering, as they induce size-dependent segregation of receptor-ligand complexes away from large phosphatases, such as CD45, thus allowing efficient phosphorylation of receptor-associated signalling motifs and initiation of signalling 14. CD45 plays an important role in lymphocyte receptor signalling, and CD45 deficient mice display severe impairment of T- and B-cell development 15. CD45 deficient mice have elevated numbers of NK cells, and these NK cells are competent in killing but not cytokine production 16,17. In order to determine whether the reduced NK-cell activation observed in response to RMA cells expressing the elongated forms of H60a was a result of decreased segregation away from the CD45 phosphatase, we tested the functional consequences of ligand elongation using CD45^−/−^ NK cells. If H60a elongation reduces NK-cell activation solely because it decreases segregation of the engaged NKG2D from the CD45 phosphatase, then H60a elongation should have no effect on the activation of CD45^−/−^ NK cells. When CD45^−/−^ NK cells were used as effectors in killing assays with RMA cells expressing the standard or elongated H60a molecules as the targets, we observed that elongation of H60a reduced lysis by these NK cells in the same manner as for CD45 positive WT NK cells (Fig. 5), demonstrating that the effects of ligand elongation were not solely caused by reduced segregation of CD45 from NKG2D.

### Ligand elongation reduces inhibition of NK-cell functions

**Figure 6:**
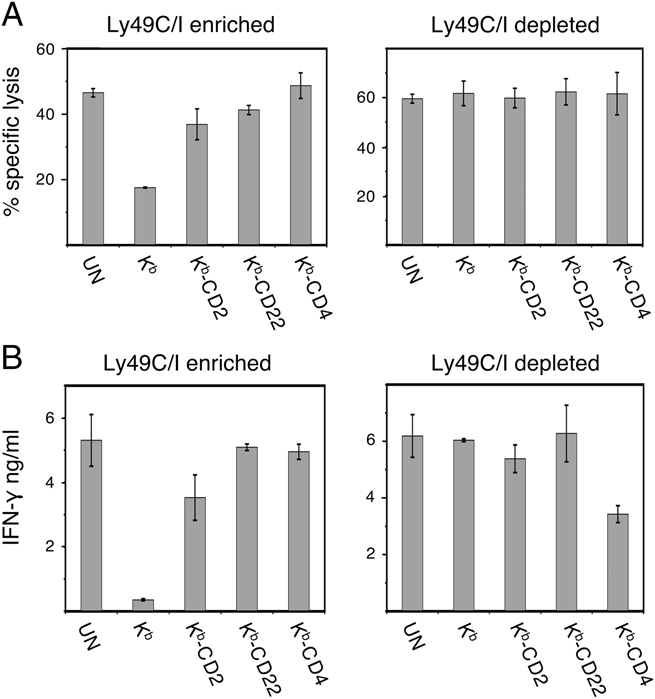
Elongation of the H-2K^b^ molecule reduces inhibition of Ly49C/I-enriched NK cells. Untransfected CHO cells (UN), or CHO cells expressing the standard K^b^OVA SCT or its elongated forms K^b^OVA SCT-CD2, K^b^OVA SCT-CD22 or K^b^OVA SCT-CD4 were used as targets for IL-2-expanded Ly49C/I-enriched and Ly49C/I-depleted NK cells in (A) cytotoxicity (effector to target ratio 20:1) and (B) IFN-γ release assays (effector to target ratio 1:5). Data show mean+SD (*n*=4) and are representative of six independent experiments.

**Figure 7:**
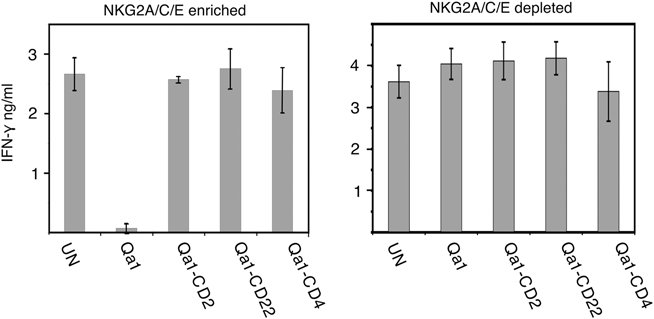
Elongation of the Qa1^b^ molecule reduces inhibition of cytokine release. CHO cells expressing the standard Qa1 SCT or its elongated forms Qa1 SCT-CD2, Qa1 SCT-CD22 or Qa1 SCT-CD4 were used as targets with IL-2-expanded CD94/NKG2A/C/E-enriched and CD94/NKG2A/C/E-depleted NK cells in IFN-γ release assays, at an effector to target ratio of 1:5. Data show mean+SD (*n*=4) and are representative of three independent experiments.

Relatively small ligand size is critical for T-cell 2,3,5 and NK-cell activation, as shown here. Because NK-cell inhibitory receptors are relatively small molecules that recognise equally small MHC class I molecules, we investigated whether ligand dimensions play a role in regulation of inhibition of NK-cell functions. The outcomes of NK-cell activating and inhibitory signalling are dramatically different, but the initiation of both types of signalling depends on tyrosine phosphorylation of receptor-associated signalling motifs. Therefore, it is plausible to suggest that NK-cell activating and inhibitory receptors may share a common triggering mechanism that depends on the small dimensions of receptor-ligand complexes. We used previously described 2 CHO cells expressing H-2K^b^ molecules and their elongated forms, because the mouse inhibitory Ly49C and Ly49I receptors recognise H-2K^b^ molecules, and untransfected CHO cells were shown to activate B6 NK cells 18. The H-2K^b^ molecules were expressed in the form of a single chain trimer (K^b^OVA SCT), consisting of the H-2K^b^ heavy chain, β_2_-microglobulin and the OVA peptide fused together with flexible linkers. Three elongated forms of K^b^OVA SCT were used: K^b^OVA SCT-CD2, K^b^OVA SCT-CD22 and K^b^OVA SCT-CD4, containing an additional two, three or four Ig domains in their membrane-proximal region, respectively 2. CHO cells expressing similar levels of the standard or elongated K^b^OVA SCT constructs were used as targets in NK-cell functional assays. B6 NK cells were sorted magnetically into Ly49C/I-enriched (approximately 75–80% Ly49C/I-positive cells) and Ly49C/I-depleted (approximately 95% Ly49C/I-negative cells) subsets, expanded in IL-2 and used as effectors. We could not compare directly inhibition of NK-cell functions mediated by K^b^OVA SCT with conventional H-2K^b^ heavy chain, because expression of H-2K^b^ heavy chain (but not of K^b^OVA SCT) in CHO cells decreased cell surface expression of the hamster MHC class I molecule Hm1-C4 (data not shown), which is the major activating ligand for B6 NK cells 19. Expression of K^b^OVA SCT on CHO cells inhibited both lysis and cytokine production by Ly49C/I-enriched, but not Ly49C/I-depleted NK cells (Fig. 6), demonstrating that K^b^OVA SCT can be recognised by Ly49C and/or Ly49I. Elongation of K^b^OVA SCT molecules resulted in decreased inhibition of both lysis and cytokine secretion by Ly49C/I-enriched NK cells (Fig. 6), but had no effect on the functions of Ly49C/I-depleted NK cells, suggesting that small ligand dimensions are important for inhibition of NK-cell functions. To confirm this finding, we generated a Qa1^b^ SCT presenting the Qdm peptide, and three elongated forms: Qa1SCT-CD2, Qa1SCT-CD22 and Qa1-CD4, analogous to the three elongated forms of K^b^OVA SCT 2. These constructs were expressed in CHO cells and the transfectants were FACS-sorted to give very similar levels of cells surface expression of the Qa1^b^ molecule (data not shown). NK cells were magnetically sorted into CD94/NKG2A/C/E-enriched (greater than 95% purity) and CD94/NKG2A/C/E-depleted (approximately 90% purity) cells and expanded in IL-2. Expression of Qa1 SCT did not inhibit lysis by CD94/NKG2A/C/E-enriched NK cells (data not shown), but strongly inhibited IFN-γ secretion by CD94/NKG2A/C/E-enriched, but not CD94/NKG2A/C/E-depleted NK cells (Fig. 7). Elongated forms of Qa1 SCT did not inhibit IFN-γ secretion by CD94/NKG2A/C/E-enriched NK cells (Fig. 7), confirming that small ligand dimensions are important for inhibition of NK-cell functions.

## Discussion

Previously, we have demonstrated the importance of small ligand dimensions for CD8^+^ T-cell activation by experimental elongation of MHC class I molecules 2,3. In the present study, similar elongation of ligands for the activating NKG2D and the inhibitory Ly49C and/or Ly49I and CD94/NKG2A receptors was found to reduce NK-cell activation and inhibition, respectively. The observed decrease in NK-cell activation was not caused by altered receptor binding affinity for the engineered H60a molecules, because target cells expressing the standard and elongated forms of ligands displayed comparable levels of staining with soluble NKG2D tetramers and supported similar levels of conjugation with NK cells. An important issue is whether the inserted Ig spacer domains are inert and only act as spacers, or could in themselves be having an effect. We chose to use exactly the same Ig domains used in our previous work on increasing the dimensions of MHC class I molecules 2,3. It seems unlikely that if the spacer domains were having functional effects they could cause both a decrease of NK-cell activation (H60a experiments), and an apparent increase of NK-cell activation (H-2K^b^ and Qa1 experiments). We have also demonstrated directly that single chain versions of MHC class I molecules can be recognised by receptors on NK cells to inhibit both cytotoxicity and cytokine production, emphasising the utility of these constructs. Similar MHC class I SCT constructs were used previously by others to investigate NK-cell education and function 20,21. Our findings suggest that NK-cell activating and inhibitory receptors may share a common mechanism of receptor triggering that depends on relatively small ligand size, and are consistent with the hypothesis that ectodomain size-dependent protein segregation at the cell contact interface plays a role NK-cell receptor triggering, as proposed by the kinetic-segregation model 14.

The kinetic-segregation model of receptor triggering proposes that during lymphocyte target cell interactions, exclusion of proteins with large ectodomains, such as the CD45 and CD148 tyrosine phosphatases, from close-contact zones formed by interacting receptor and ligand pairs of small dimensions, shifts the balance of constitutive phosphorylation and dephosphorylation towards phosphorylation, allowing receptor triggering 14. Some previously described features of NK-cell receptor signalling indicate that the model may be applicable to NK cells. Signalling *via* NK-cell activating and inhibitory receptors is initiated by tyrosine phosphorylation of receptor-associated cytoplasmic sequences. Murine NKG2D associates with the DAP10 and DAP12 adaptor molecules, whereas Ly49C, Ly49I and NKG2A contain an ITIM sequence in their cytoplasmic tails. Treatment with pervanadate, a protein tyrosine phosphatase inhibitor, results in DAP10 phosphorylation 22,23 and in Ly49C phosphorylation 24 in unstimulated NK cells. This suggests that the NK-cell receptor signalling motifs are being constitutively phosphorylated and dephosphorylated, even in the absence of receptor ligation, although it is possible that pervanadate may also activate some tyrosine kinases. Upon ligand binding, this phosphorylation and dephosphorylation balance must change in favour of tyrosine phosphorylation. The phosphatases mediating NK-cell receptor associated motif dephosphorylation have not as yet been identified. However, there is some evidence that NK-cell receptor signalling motifs are substrates for CD45, as DAP12 is hyperphosphorylated in CD45-deficient NK cells 25. However, elongation of H60a reduced activation of CD45^−/−^ NK cells (Fig. 5), demonstrating that the effects of ligand elongation observed here were not solely caused by reduced segregation of CD45 from NKG2D. However, there remains a possibility of size-dependent segregation from other phosphatases with large ectodomains. Electron microscopy examination of human NK cells interacting with target cells expressing a ligand for the inhibitory receptor KIR2DL1 revealed the presence of wide and narrow regions between the apposing membranes, and accumulation of the ligand in the narrow regions, as predicted by the kinetic-segregation model 26. Moreover, phosphorylated KIR2DL1 molecules were observed to localise in microclusters during inhibitory interactions 27.

The relatively small dimensions of NK-cell activating and inhibitory receptor–ligand complexes may facilitate their size-dependent co-localisation at the NK-cell immunological synapse, thus facilitating signal integration. Co-localisation of human inhibitory and activating NK-cell receptors was demonstrated previously for the activating receptors CD2 and 2B4, and inhibitory KIR2DL1/2 receptors 28. It is not yet known whether efficient inhibition of NK-cell activating signalling requires co-localisation of the activating and inhibitory receptors 29. Phosphorylated ITIM sequences can recruit the SHP-1, SHP-2 and SHIP phosphatases, which then dephosphorylate, directly or indirectly, several proteins involved in the activation of NK-cell responses. Critically, SHP-1 and SHP-2 are active only when bound to the ITIM motifs, as this releases the phosphatase catalytic domain from an inhibitory interaction with the N-terminal SH2 domain. Therefore, NK-cell inhibition is spatially restricted and is predicted to be efficient only if the engaged activating and inhibitory receptors are localised within relative proximity. The reduction of inhibition of NK-cell functions observed here after elongation of the H-2K^b^ or Qa1^b^ molecules may result from size-dependent segregation of the small, unaltered activating complexes from the experimentally elongated inhibitory complexes. Further work investigating the functional consequences of the simultaneous elongation of ligands for both activating and inhibitory receptors is required to test this possibility.

Our results suggest that size-dependent protein segregation at the interface between NK cell and target cell is critical for controlling NK-cell activation, as experimental elongation of ligands for NKG2D or Ly49C and/or Ly49I resulted in a decrease of NK-cell activation or inhibition, respectively. We propose that size-dependent protein segregation is a general mechanism that initiates and/or facilitates lymphocyte receptor triggering. Some activating and inhibitory NK-cell receptors are also expressed on other cell types. NKG2D can be expressed on activated CD8^+^ T cells, subsets of γδ T cells and NKT cells 12. Ly49C/I can be expressed on subsets of T cells 30, NKT cells 31 and B-1 cells 32. The general principle of size-dependent protein segregation should also apply to triggering of these NK-cell receptors on other cell types. A range of NK-cell receptors that can signal *via* ITAM or ITIM-containing cytoplasmic sequences have been described; however, ligands for some of them have not yet been identified. Based on the work presented here, we predict that these as yet unidentified NK-cell ligands have relatively small ectodomains. A requirement for small ligand dimensions in effective regulation of NK-cell function has important implications for the identification of natural ligands for NK-cell receptors, and similarly for the manipulation of NK-cell receptors/ligands for possible therapeutic purposes.

## Materials and methods

### Antibodies

The following mAb were used, obtained from BD Pharmingen unless indicated otherwise: anti-H60 clone 205326 (R&D Systems), anti-Qa1^b^ clone 6A8.6F10.1A6, anti-Ly49C/I clone 5E6, anti-NKG2A/C/E clone 20d5, anti-H-2K^b^ clone CTKb (AbD Serotec), anti-CD49b clone DX5 and anti-NK1.1 clone PK136.

### Recombinant DNA constructs

Standard molecular biology techniques were used. A unique *BamH*I restriction site was introduced into the H60a sequence cloned into the pKG4 mammalian expression vector using the forward 5′caaatgccactcagctggatccacagtgaataacttc 3′ and the reverse 5′gaagttattcactgtaggatccagctgagtggcatttg 3′ oligonucleotides by directed mutagenesis using a QuickChange mutagenesis kit (Stratagene), according to the manufacturer's instructions. Inserts encoding domains 1 and 2 of human CD2 (for H60a-CD2), or domains 1–4 of human CD4 (for H60a-CD4), were excised from the previously described equivalent elongated K^b^OVA SCT constructs 2, and ligated into the new *BamH*I site in the H60a cDNA. A new Qa1^b^ SCT presenting the Qdm peptide (AMAPRTLLL), and the three elongated forms of Qa1^b^ SCT were generated exactly as described previously for the K^b^OVA SCT molecules 2, using an introduced unique *BamH*I restriction site.

### Cell cultures and transfection

RMA cells were cultured in RPMI 1640 medium with 2 mM glutamine and 10% FBS. RMA cells were transfected by electoporation (Amaxa Biosystems, nucleofection kit L, program C-1). Stable RMA transfectants were selected using 1 mg/mL Geneticin (G418 sulphate). CHO cells were cultured in nutrient mixture F-12 (Ham) with 4 mM glutamine and 5% FBS. CHO cells were transfected using poly-l-ornithine 33. Stable CHO transfectants were selected using 0.8 mg/mL Geneticin. Transfectants were FACS-sorted (FACSDiva, BD) for cell surface expression of the relevant molecule. All media and supplements were from Invitrogen.

### NKG2D tetramer staining and cellular conjugation assay

In total, 5×10^5^ cells were stained with PE-conjugated NKG2D tetramers 34 diluted 1:100 in PBS/1% BSA. Following 30 min incubation on ice, cells were washed and analysed by flow cytometry (CyAn™, Beckman Coulter). To investigate conjugate formation, NK and RMA cells were labelled with 4 μM PKH26 and PKH67 membrane dyes (Sigma), respectively, according to the manufacturer's instructions. PKH26 and PKH67 are detected on the flow cytometer channels FL2 and FL1, respectively. Following extensive washing, the cells were rested for 1 h at 37°C. Labelled NK and target cells were mixed at a 1:2 ratio and incubated at 37°C. After 10 min incubation, the cells were fixed using Cytofix (BD), gently resuspended and analysed immediately by flow cytometry. Quadrants were set to define FL1^high^, FL2^high^ and FL1^high^/FL2^high^ cell populations. The percentage of FL1^high^/FL2^high^ events relative to total events was taken as an estimate of conjugate formation.

### NK-cell isolation and culture

Spleens were harvested from 8–10 wk-old female C57BL/6 mice (Harlan). NK cells were isolated from splenocyte suspensions by magnetic separation using a Mouse NK Cell Isolation kit (Miltenyi Biotec). This allowed the isolation of untouched NK cells by positive depletion of other cell types. When required, freshly isolated NK cells were stained with PE-conjugated antibody for the receptor of interest and subjected to magnetic separation into the positive and negative populations, using anti-PE microbeads (Miltenyi Biotec). Anti-Ly49C/I (clone 5E6) and anti-NKG2A/C/E (clone 20d5) antibodies were used in this way. NK cells were cultured in RPMI 1640 with 2 mM glutamine and 10% heat-inactivated FBS, 20 μM 2-mercaptoethanol, 1 mM sodium pyruvate, 1 mM MEM non-essential amino acids and 1000 U/mL human recombinant IL-2 for 5 days. NK cells from CD45^−/−^ mice 15 on the B6 background were isolated and cultured using the same method. The purity of NK-cell cultures was routinely monitored by anti-CD49b (clone DX5) and anti-NK1.1 (clone PK136) antibody staining and flow cytometry.

### *In vitro* functional assays

NK-cell cytotoxicity was measured using a standard 5-h ^51^Cr release assay. The levels of spontaneous ^51^Cr release from target cells were below 10% in the assays. To investigate IFN-γ secretion, 5×10^4^ NK cells were incubated with target cells at a 1:5 NK:target cell ratio for 24 h. Supernatant was collected and frozen for at least 24 h prior to analysis using standard IFN-γ ELISA. GraphPad software was used to perform tests of statistical significance.

### *In vivo* killing assay

The two populations of RMA cells to be tested were labelled separately with 2 μM PKH26 and PKH67 membrane dyes, according to the manufacturer's instructions. Following extensive washing, the labelled cells were rested for at least 1 h at 37°C. The cells were then re-counted, mixed at a 1:1 ratio, and 10^6^ cells in total resuspended *per* 0.8 mL sterile PBS. An aliquot of the cell suspension was analysed by flow cytometry immediately to verify the 1:1 ratio of the two cell populations. The cell suspension was injected i.p. into B6 mice with 0.4 mL *per* mouse and two to four mice *per* group. A separate aliquot of the same cell suspension was cultured *in vitro* (in the absence of NK cells) for later. 48 h after the injections, the mice were culled and the peritonea washed out with PBS. The peritoneal lavage, and the equivalent *in vitro* culture were then both analysed by flow cytometry. All animal experiments were carried out in accordance with UK Home Office requirements.

## Abbreviation

SCT: single chain trimer
